# Critical appraisal of “A cross-sectional study on the effects of intravesical BCG on urinary microbiota in bladder cancer patients”

**DOI:** 10.1097/MS9.0000000000003953

**Published:** 2025-09-23

**Authors:** Haseeb Safdar Ali, Esha Bilal, Huzaifa Noor, Muddassir Khalid, Supoma Ghosh Ria

**Affiliations:** a Nishtar Medical University, Multan, Pakistan; b Mugda Medical College, University of Dhaka, Dhaka, Bangladesh

*Dear Editor,*

We read with great interest the recent work of Li *et al* concerning alterations in the urine microbiota after intravesical BCG treatment of patients with bladder cancer^[1]^. The authors reveal some characteristics of microbial patterns unique to the treatment groups and indicate that BCG might have specific treatment effects on the urinary microbiota through promoting metabolic pathways. Nevertheless, some methodological and interpretive aspects should be elaborated to ensure the study receives more clinical and scientific credibility.

First, there is a concern with the categorization of the surveillance group as a group of recovered bladder cancer patients. Non-muscle-invasive bladder cancer (NMIBC) has a high rate of recurrence, up to 70% after 5 years, which necessitates lifelong monitoring^[2]^. Recording such patients as recovered may be confusing to the reader and may lead to an interpretational bias. Also, the meaning of the surveillance group is not consistent throughout the text; in Methods, it is called recovered, but in Results, it is phrased as not having BCG instillation. Uniform, clinically specific nomenclature would help increase clarity and cut the chances of misunderstanding.

Second, valuable baseline demographic factors are partly unreported; this reduces the efficacy of between-group comparisons. For example, there is no data regarding the body mass index of the control group, despite providing a *p* value. Some participants lack data about smoking status across different groups, yet they are used to make statistical comparisons without any remarks. The age is recorded in the form of a mean (median) without the mean data points. It is essential to be clear about missing data in observational research, including the extent of the missing data and how it was dealt with. Strict observation of such established guidelines as STROBE or the TAR-MOS framework^[3,4]^ would improve methodological transparency, analytical validity, and reproducibility.

Lastly, the example cited in the discussion against the rising rates of Pseudomonas in the post-BCG group population scarcely compares to the antitumor effects of oportuzumab monatox. It could be misleading since it could confuse the reader. Although the therapeutic value of oportuzumab monatox is recognized in the treatment of refractory NMIBC, it is not a microbial colonization of the urinary tract but, instead, a recombinant immunotoxin, a conjugate of an antibody to EpCAM fused to Pseudomonas exotoxin A^[5]^. Separating the biological origin of the toxin and the real presence of microbes would aid in preventing a possible misinterpretation of the microbiota-drug relationships.

Altogether, the research by Li *et al* is of importance given the growing body of evidence that describes the role of a cancer microbiome, investigating the effects of the BCG treatment on bladder cancer. Clarifying the issues surrounding definitions of patient groups, missing elements in the baseline, and analysis of microbiota and drug interactions would also improve the study’s clarity, clinical aspects, and reproducibility. The refinements will be crucial since the field will continue to transform itself with the application of microbiome knowledge into uro-oncological practice.

TITAN Guidelines: This manuscript complies with TITAN Guidelines, 2025, declaring no use of AI^[6]^.

## Data Availability

Not applicable.

## Research registration unique identifying number (UIN)

Not applicable for this letter to the editor.
